# Supplementary material for: Allelopathic and auto-toxic effects of aqueous extracts of Codonopsis pilosula root secretions
Source: PLoS One. 2025 Jan 31;20(1):e0315657. doi: 10.1371/journal.pone.0315657 (PMC11785337; doi:10.1371/journal.pone.0315657)
Supplement: Attachment — Submitted filename: Response to Reviewers.(1).docx [file pone.0315657.s001.docx]

**Response to Reviewers**

To Reviewers:

Thank you for your letter and the constructive comments on this article in your busy schedule. All of us authors have carefully read the comments that you have given us, and have discussed and revised each of these issues. The following is my list of revisions. In addition. we have resubmitted a new manuscript in the revised state, with the revisions highlighted in red. If there are any incorrect answers or questions in the manuscript. please do not hesitate to let us know.

**Abstract**

1. For a broader application of findings, the abstract should include a clearer statement.

We appreciate the reviewer's suggestion for a clearer statement in the abstract to enhance the broader application of our findings. We have revised the abstract as follows:

"Water-soluble constituents in the root exudates of *C. pilosula* exhibit both allelopathic and auto-toxic properties, significantly impeding its growth. This study investigated the impact of aqueous extracts from root exudates at different developmental stages on seed germination and seedling growth to address the issue of continuous cropping constraints. We isolated and characterized significant auto-toxic allelochemicals. Our findings revealed that the inhibitory effects on seed germination, seedling growth, and the activity of antioxidant enzymes-collectively referred to as a comprehensive effect-intensified progressively with increasing concentrations of the aqueous extracts from *C. pilosula* root exudates . The most pronounced auto-toxic comprehensive effect was observed with a 50 mL extract during the reproductive phase, reducing SOD and POD activities and MDA levels in *C. pilosula* seedlings by 72.3%, 71.7%, and 83.3% respectively compared to the control (*P* < 0.05). Three main allelopathic compounds - acetic acid, hexamethyl cyclotrisiloxane, and methoxybenzene oxime - were isolated and identified in the aqueous extracts. Acetic acid, accounting for 44.1% of the total chemical profile, had a significant inhibitory effect on seed germination and seedling growth (*P* < 0.05). These findings provide valuable insights for developing strategies to mitigate the negative effects of allelopathy and auto-toxicity in *C. pilosula* cultivation, thereby promoting sustainable production."

This revised abstract now clearly emphasizes the application potential of our research findings in the context of *C. pilosula* cultivation. We hope it meets the reviewer's expectations and provides a more comprehensive and useful summary of our study.

2. Please rewrite some lengthy sentences into shorter ones for better understanding

Thank you for your valuable suggestion. We have rewritten some of the lengthy sentences to make the content more understandable.

For example, we divided the sentence "Water-soluble constituents in the root exudates of C. pilosula exhibit both allelopathic and auto-toxic properties, which substantially impede its growth" into "Water-soluble constituents in the root exudates of C. pilosula have both allelopathic and auto - toxic properties, which greatly hinder its growth". This makes the idea more straightforward and easier to follow.

Similarly, we broke down other complex sentences to enhance the clarity and readability of the text. We believe these changes will improve the overall quality of the manuscript and make it more accessible to the readers.

We appreciate your careful review and the opportunity to improve our work.

3. Specific details about the methodology (e.g., concentration of extracts) are not necessary in the abstract.

We sincerely appreciate your comment. We understand your perspective that specific details about the methodology, such as the concentration of extracts, may not typically be necessary in the abstract. However, in our case, the fact that the extract at a concentration of 50 mL showed the most pronounced auto-toxic comprehensive effect is not merely a methodological detail.

This finding is a central and distinctive outcome of our study. It is not just about the concentration per se but rather a significant discovery that helps to define the nature and extent of autotoxicity. By including this information, we aim to provide a more comprehensive and accurate understanding of our research at first glance for the readers.

We have considered your suggestion carefully and will further reflect on how to present this information in the abstract in the most appropriate way. We may explore alternative phrasings to convey this key finding without over-emphasizing the methodological aspect.

Thank you again for your valuable feedback, which has helped us to improve our manuscript.

**Introduction**

1. Streamlining some background information to avoid redundancy in the introduction.

Thank you very much for your valuable comments. Regarding the issue you pointed out of streamlining the background information in the introduction to avoid redundancy, we have conducted in-depth reflection and careful revision. During the revision process, we carefully sorted and screened the background information in the introduction. We deleted some repeatedly stated content and retained the background knowledge closely related to the research topic and necessary. First, we introduced *Codonopsis pilosula* medicinal materials. Secondly, we used specific examples of different plants to describe the universality and severity of continuous cropping obstacles. Finally, we focused on the relevant content of *Codonopsis pilosula* rhizosphere secretion and draw out the importance and purpose of this study.

2. It is necessary to elaborate on some references to clarify their relevance to the current research.

Thank you very much for your valuable comments. Regarding the references [7], [8], and [9], here is the elaboration:

Reference [7] on the accumulation of reactive oxygen species (ROS) induced by autotoxins at the root tip of *Codonopsis pilosula* is highly relevant as it provides crucial insights into an important factor affecting plant toxicity. This understanding helps to establish a foundation for further research on the overall health and growth of *Codonopsis pilosula* and potentially informs strategies to mitigate toxicity.

Reference [8] by Wang et al. which studied the effects of water extracts of *Codonopsis pilosula* straw on the seed germination and seedling growth of three traditional Chinese medicines is significant. This reference shows the potential allelopathic effects of *Codonopsis pilosula* straw on other species, highlighting the complex interactions within the ecosystem of traditional Chinese medicine plants. It also provides a basis for considering the impact of *Codonopsis pilosula* cultivation on neighboring plants and vice versa.

Reference [9] by Ren et al. on the allelopathic effects of root aqueous extracts of *Codonopsis pilosula* at different growth stages on multiple crops is essential. It further expands on the allelopathic properties of *Codonopsis pilosula* and shows how these effects vary with growth stages and concentration. This knowledge is valuable for understanding the ecological planting and sustainable cultivation practices of *Codonopsis pilosula*)

In conclusion, these references play a crucial role in supporting and enriching the current research, providing both theoretical and practical implications for the study of *Codonopsis pilosula* and its interactions with other plants.

**Methodology**

1. To improve readability, rewrite the methodology section, as it is very dense and may break into several sub-sections.

Thank you for your valuable comments and suggestions. We have made the following revisions to address your concerns.

1. Rewriting the Methodology Section

We have rewritten the methodology section to improve readability and divided it into several sub-sections as follows:

2.1 Study Site

We provide detailed information about the location of the experiment in Tanchang County, Longnan City, Gansu Province, including its coordinates, altitude, climate characteristics, soil properties, and previous land use. This helps to establish the context for the study.

2.2 Collection and Hydroponic Culture of Codonopsis Seedlings

This sub-section describes the process of collecting well-grown and pest-free Codonopsis seedlings from the test site, cleaning and sterilizing them, and then cultivating them hydroponically. We explain the use of foam boards with uniform perforations and hydroponic tanks filled with a 1/2-strength Hoagland nutrient solution. We also provide information about the composition of the Hoagland nutrient solution and its importance for plant growth.

2.3 Preparation of Aqueous Solution of Root Secretion of *Codonopsis pilosula*

Here, we describe the collection of root secretions at three different growth stages of *Codonopsis pilosula*. We explain how the roots were cleaned, placed in sterile deionized water, and then the resulting aqueous solution was concentrated and stored for use in seed germination experiments. We also mention the repetition of the collection process and the combination of solutions from the same growth period.

2.4 Determination of Indicators and Methods

This sub-section is further divided into several parts:

2.4.1 Determination of Seed Germination Index of *Codonopsis pilosula*

We explain the use of distilled water as a control and different concentrations of root secretion aqueous leachate as treatment groups. We describe the seed sterilization process, incubation conditions, and the criteria for seed germination.

2.4.2 Determination of Growth Indexes of *Codonopsis pilosula* Seedlings

We detail the "small cup method" for cultivating seedlings and the measurement of various growth indicators such as stem length, root length, and total biomass. We also explain how total fresh weight is determined.

2.4.3 Verification of Allelopathic Effects of Monomer Compounds

We describe the identification of the most abundant monomer compounds and their preparation into different concentration solutions. We explain the seed germination and seedling growth tests used to evaluate the auto-toxic effects of these compounds and the calculation of the Williamson allelopathic response index and allelopathic comprehensive effect index.

2.4.4 Determination of Antioxidant Enzyme Activity

We explain the methods used to measure superoxide dismutase (SOD) activity, peroxidase (POD) activity, and malondialdehyde (MDA) content.

2.5 Identification of Allelopathic and Auto Toxic Substances

We describe the use of gas chromatography-mass spectrometry (GC-MS) to qualitatively identify the chemical constituents in an aqueous solution of *Codonopsis pilosula* root secretion. We provide details about the instrument used, detection conditions, and data analysis methods.

2.6 Data Processing

We specify the software used for data analysis, including IBM SPSS Statistics version 26 for one-way analysis of variance and Origin 2021 for correlation analyses and graphing.

2. For improved readability, provide a brief explanation of technical terms like "Hoagland nutrient solution".

Thank you for your valuable comments and suggestions. We have made the following revisions to address your concerns.we have provided a brief explanation of the technical term "Hoagland nutrient solution". Hoagland nutrient solution is a widely used nutrient formulation in plant cultivation. It is a carefully balanced mixture of various essential elements required for plant growth. The different concentrates in the solution provide plants with important macronutrients like nitrogen (from calcium nitrate and ammonium phosphate), phosphorus (from ammonium phosphate), potassium (from potassium nitrate), calcium (from calcium nitrate), and magnesium (from magnesium sulphate), as well as micronutrients like iron (from ferrous sulphate heptahydrate). The specific ratio of these components in the solution is designed to meet the nutritional needs of different plants at various stages of growth.

**Results**

1. Please highlight the most critical findings; it could be better to understand your work.

We are extremely grateful for your suggestion. We have made revisions to the results section and highlighted the key findings. As follows are the key discoveries:

3.1 Effects of aqueous extracts of root secretions of *C. pilosula* on own seed germination and seedling growth at different growth stages.

The water extracts of root exudates of *Codonopsis pilosula* at different concentrations have an inhibitory effect on the seed germination and seedling growth of *Codonopsis pilosula* . Moreover, at different growth stages, it showed a trend of “weak in the initial stage, enhanced in the reproductive stage, and weakened in the harvest stage”. At the same time, the concentration of the extract is positively correlated with the inhibitory effect.

The |Ir| value of the germination rate of *Codonopsis pilosula* seeds was less than that of the germination potential, indicating that the water extract had a greater impact on the germination potential.

The stem length, root length, and total fresh weight of *Codonopsis pilosula* seedlings continuously decreased with the increase of the concentration of the water extract. Among them, the water extract concentrated at 50 mL had the most significant inhibitory effect, indicating that the concentration of root exudates is positively correlated with the degree of inhibition of seedling growth. In actual planting, it is necessary to consider controlling the accumulation of root exudates to promote growth.

Based on comprehensive analysis of various growth indicators, the comprehensive allelopathic effect index was negative. The autotoxic effect of *Codonopsis pilosula* increased with the increase of the concentration of the water extract. The root exudate water extract of *Codonopsis pilosula* concentrated at 50 mL during the reproductive stage had the most significant inhibitory effect and the strongest autotoxic comprehensive effect.

3.2 Effects of aqueous extracts of root secretions of *C. pilosula* on antioxidant enzyme activities own seedlings at different growth stages.

Different concentrations of aqueous extracts of *Codonopsis pilosula* root secretions significantly reduced the activity of reactive oxygen scavengers in *C. pilosula* seedlings at different growth stages, leading to a large accumulation of lipid peroxidation products.

The activities of superoxide dismutase (SOD) and peroxidase (POD) decreased with increasing concentrations of the extracts. The 50 mL concentrated extract had the most significant reduction in POD activity during the reproductive stage (71.7% lower than the control) and the weakest SOD activity was observed under the same concentration during the propagation period.

The 50 mL concentrate also led to the highest accumulation of malondialdehyde (MDA) content in the leaves, especially during the reproductive stage with an 83.3% increase compared to the control.

3.3 Identification of allelopathic components in the aqueous extract of root exudates of *C. pilosula*.

We identified five main components in the root exudates, with acetic acid having the highest relative content at 44.1%, followed by hexamethylcyclotrisiloxane and methoxybenzene oxime at 22.3% each. The remaining components have less than 10% relative content.

The three components with the highest content, namely acetic acid, hexamethylcyclotrisiloxane, and methoxybenzene oxime, exhibit allelopathic effects.

3.4 Validation of allelopathic effects of different concentrations of acetic acid.

Acetic acid inhibits seed germination and seedling growth of *Codonopsis pilosula*, with a significant concentration gradient effect.

As acetic acid concentration increases, germination rate, germination potential, stem length, root length, and total fresh weight of *Codonopsis pilosula* seedlings decrease significantly.

High concentrations of acetic acid strongly inhibit POD and SOD activities in *Codonopsis pilosula* seedling leaves, and at 1 g/L, the inhibition rate is 100%, completely destroying the protective enzyme system.

MDA content in *Codonopsis pilosula* seedlings accumulates with increasing acetic acid concentration, reaching the highest at 1 g/L, indicating enhanced membrane lipid peroxidation and greater damage to leaf cell membranes.

2. Figure 1B for germination force does not show a bar of 50 mL at the middle growth stage; please clarify.

We are very sorry for our careless mistake. Thank you for the heads up. We have made a correction to Figure 1B

3.In the results section, brief interpretations of the results could help connect the data to the study's objectives.

Thank you for your valuable suggestion. We have added brief interpretations of the results in the results section as follows:

The allelopathic indices of different concentrations of leaching solutions are all negative, indicating that the leaching solution of *Codonopsis pilosula* root secretions has an inhibitory effect on seed germination and seedling growth, and the autotoxic effect increases with the increase of the concentration of the aqueous solution.

As the concentration of the aqueous extract increases, the stem length, root length, and total fresh weight of *Codonopsis pilosula* seedlings continuously decrease, indicating that the concentration of root exudates is positively correlated with the degree of inhibition of seedling growth.

The increase in malondialdehyde (MDA) content, especially when using 50 ml of concentrated solution during the reproductive period, indicates that the aqueous extract of *Codonopsis pilosula* root secretions can destroy the antioxidant system of seedlings, leading to an increase in lipid peroxidation.

The inhibition rate of POD and SOD activities of *Codonopsis pilosula* seedlings by 1 g/L acetic acid reaches 100%, indicating that 1 g/L acetic acid will completely damage the protective enzyme system of *Codonopsis pilosula* leaves and make the *Codonopsis pilosula* plants lose their antioxidant capacity.

Under the treatment of 0.01 g/L and 0.1 g/L acetic acid, the MDA content of *Codonopsis pilosula* seedlings accumulates significantly. When the acetic acid concentration rises to 1 g/L, the MDA content accumulates to the highest level, indicating that the increase in acetic acid concentration will increase the degree of membrane lipid peroxidation in the tissues or organs of *Codonopsis pilosula* seedlings and increase the degree of damage to the cell membranes of leaves.

These results help us better understand the impact of root secretions and acetic acid on *Codonopsis pilosula* growth and provide important insights for further research on mitigating these negative effects.

4.It is advisable to include the structure of the identified compounds in order to enhance the appeal of the work to the readers.

Thank you for your valuable suggestion. We agree that including the structures of the identified compounds can enhance the attractiveness of our work to readers. We will make efforts to incorporate the structures of the three compounds with the highest contents—acetic acid (C₂H₄O₂), hexamethyl cyclotrisiloxane (C₆H₁₈O₃Si₃), and methoxybenzene oxime (C₈H₉NO₂) - in an appropriate way in our revised manuscript. This will not only make the content more visually appealing but also help readers better understand the chemical nature of these compounds and their potential roles in the processes we are studying.

Acetic acid	Hexamethyl cyclotrisiloxane	Methoxybenzene oxime

**Discussion**

1. To avoid repetition, rewrite the condensed discussion section.

Thank you for your suggestion regarding avoiding repetition in the discussion section. We have carefully re-evaluated and rewritten the discussion section to eliminate any redundancies with the results section.

We have taken great care to ensure that the discussion focuses on the interpretation, analysis, and implications of the results rather than simply restating them. By referring back to the results in a more integrated and meaningful way, we aim to provide a more in-depth understanding of the significance of our findings.

We have rephrased and reorganized the content to highlight the key points and connections, while also drawing comparisons with previous studies and identifying potential areas for future research. We appreciate your feedback and believe that these changes will better meet the standards of a well-structured and comprehensive scientific paper.

2. Explain thoroughly the role of allelopathy in specific compounds and their modes of action.

We sincerely appreciate your valuable comment and the opportunity to further clarify the role of allelopathy in acetic acid and its modes of action.

Allelopathy plays a crucial role in the interaction between plants and the environment. In the case of acetic acid, it exerts significant harmful effects on plants. Acetic acid causes membrane leakage in plant cells. This disrupts the intracellular material balance as essential substances leak out, interfering with the normal metabolic pathways of the cells. The damage to the cell structure also affects the function of organelles, further disrupting the normal physiological processes within the cells. Simultaneously, the prevention of cell absorption of water and nutrients due to membrane damage has a profound impact on plant growth and development.

In addition, the loss of root ions caused by acetic acid disrupts the ionic balance within the plant. This interference in numerous metabolic processes leads to a decrease in the plant's ability to resist stress. As the concentration of acetic acid increases, the accumulation of MDA content gradually rises (as shown in Table 1). This indicates that acetic acid induces lipid peroxidation of the plant cell membrane.

The mode of action of acetic acid can be explained as follows: Firstly, acetic acid can interact with the components of the cell membrane, altering its physical and chemical properties. This interaction leads to an increase in membrane permeability and the leakage of intracellular substances. Secondly, the accumulation of reactive oxygen species (ROS) due to the disruption of cellular metabolism by acetic acid attacks the unsaturated fatty acids in the cell membrane, initiating lipid peroxidation. The products of lipid peroxidation, such as malondialdehyde (MDA), can cross-link with membrane proteins and phospholipids, further damaging the membrane structure and function. This damage not only affects the selective permeability of the membrane but also disrupts the normal operation of ion channels and transport proteins, thereby interfering with the absorption and transport of water and nutrients.

In conclusion, our study has demonstrated the harmful effects of acetic acid on plants through multiple aspects and has explained the mode of action at the cellular and molecular levels. We will continue to conduct in-depth research in this area to provide a more comprehensive understanding of the role of allelopathy in plant-plant interactions.

Thank you again for your valuable suggestions and comments, which have greatly contributed to the improvement of our research.

3. It is very important to use it in wider agricultural practices. We could elaborate on the study's relevance by focusing on its application beyond *C. pilosula*.

We sincerely appreciate your suggestion regarding the application of our study in wider agricultural practices. We are fully aware of the importance of extending the implications of our research beyond Codonopsis pilosula. The study on the allelopathic self-toxicity of Codonopsis pilosula has indeed provided valuable insights into agricultural practices. The understanding of allelopathy can guide crop rotation and intercropping strategies. By recognizing the allelopathic effects of different plants, we can better plan the sequence and combination of crops in the field. This not only helps to improve soil fertility but also reduces the occurrence of pests. The allelochemicals in root exudates play a crucial role in influencing the soil microbial community and soil fertility. Our research has shown that by understanding these effects, we can take measures to regulate the allelochemicals. This, in turn, can lead to the improvement of soil quality. For example, we can explore ways to enhance the beneficial effects of certain allelochemicals on soil microorganisms while minimizing the negative impacts. In conclusion, our research on the allelopathic self-toxicity of Codonopsis pilosula has far-reaching implications for agricultural sustainability and the health of the agricultural ecosystem. We will further elaborate on these applications in the revised manuscript to make the study's relevance more prominent and applicable in a broader agricultural context. Thank you again for your valuable comment.

4. The aqueous extract of *C. pilosula* root secretions overlooked other significant factors, such as volatile compounds, soil interactions, or microbial activity. The section should acknowledge such limitations, suggesting future research to explore the role of allelopathic agents.

We sincerely appreciate your valuable comment. We fully recognize that in our study on the aqueous extract of *C. pilosula* root secretions, we have overlooked several significant factors such as volatile compounds, soil-root secretion interactions, and microbial activity.

We will definitely acknowledge these limitations in the relevant section of our manuscript. Regarding the future research, we are highly committed to exploring the role of allelopathic agents in these aspects. We will conduct in-depth studies on how volatile compounds influence the allelopathic effect. For example, we plan to identify and quantify the volatile compounds in the root secretions and investigate their effects on the growth and development of *C. pilosula* and other related plants. In addition, we will focus on how the soil-root secretion interactions change the activity of allelopathic substances. We will explore the complex relationships between different soil properties and the allelopathic substances, as well as how these interactions affect the availability and function of allelopathic agents in the soil. Moreover, we will also study the role of microbial activity in the allelopathic process. We will analyze the composition and function of the microbial community in the rhizosphere of *C. pilosula* and investigate how microorganisms interact with allelopathic substances and how they affect the allelopathic effect.

By conducting these studies, we aim to solve the existing problems and gain a more comprehensive understanding of the allelopathic mechanism of *C. pilosula* root secretions. We believe that this will not only enhance the scientific value of our research but also provide more practical guidance for agricultural production and ecological protection.

Thank you again for your insightful suggestion, which has greatly inspired and guided our future research.

**References**

1. Integrate some recent references that might be relevant to the study.

Thank you for putting forward the valuable suggestion of integrating the recent references related to the research. We have taken this comment seriously and conducted a comprehensive search for the latest literature. We have identified several recent studies that are highly relevant to our research topic. As follows:

Zhang, P., Xia, L., Sun, Y. et al. Soil nutrients and enzyme activities based on millet continuous cropping obstacles. Sci Rep 14, 17329 (2024). https://doi.org/10.1038/s41598-024-68278-x

Chen, G., Xue, Y., Yu, X., Li, C., Hou, Y., Zhu, H., & Zhang, X. (2022). The structure and function of microbial community in rhizospheric soil of american ginseng (Panax quinquefolius L.) changed with planting years. Current microbiology, 79(9), 281

Wang, F., Jun, Z., Na, J., Hong, C., Ju, Y., Dan, G., & Peng, G. (2024). Effects of Water Extracts of Codonopsis pilosula on Medicines Seeds and Their Seedlings. Journal of Henan Agricultural Sciences, 53(4), 57.

Ren, F. Y., Qiu, D. Y., Shen, P. R., Du, M. X., Chen, Y., & Hu, F. D. (2022). Allelopathic effects of aqueous extracts from Codonopsis pilosula roots. Lishizhen Medicine and Materia Medica Research, (08), 1982-1986.

Li, Y., Luo, L. F., Wang, X. D., et al. Isolation and identification of saponin-tolerant Trichoderma notoginseng rhizosphere and evaluation of its antagonistic probiotics. Journal of China. Agricultural University, 2023, 28(08): 133-143. doi: 10.11841/j.issn.1007-4333.2023.08.11

Zhe, W., Li, Y. H., et al. Advantage analysis of circulation pool method in quality evaluation of proprietary Chinese medicine: A case study of Danshen tablets. Chinese journal of traditional Chinese medicine. 2023, 1-13. doi: 10.19540 / j. carol carroll nki CJCMM. 20230529.302.

Makhwitine, J. P., Kumalo, H. M., Sizwe, N. et al. Epigenetic Induction of Secondary Metabolites Production in Endophytic Fungi Penicillium chrysogenum and GC-MS Analysis of Crude Metabolites with Anti-HIV-1 Activity. Microorganisms,2023, 11, 6. doi:10.3390/MICROORGANISMS11061404.

Lin, C., Qiu, D. Y., Chen, Y., et al. Effects of rhizosphere soil extract of *C. pilosula* on seed germination and seedling growth. Jiangsu Journal of Agricultural Sciences, 2022, 38(04): 900-906.

We believe that the integration of these recent references has enhanced the quality and relevance of our manuscript. We are confident that our revised paper will provide a more comprehensive and up-to-date view of the research topic, and will be of greater interest to the readers of the journal. Thank you again for your helpful feedback.

2. Ensure that all references follow the journal's formatting guidelines.

Thank you for bringing this to our attention. We will carefully check and ensure that all references follow the journal's formatting guidelines. We understand the importance of maintaining a consistent and proper reference format, as it enhances the readability and credibility of our research.We will go through each reference one by one, making the necessary adjustments in terms of citation style, punctuation, and other formatting details. We will also double - check the accuracy of the information provided in each reference to ensure that there are no errors or omissions.We appreciate your suggestion and will do our best to meet the journal's requirements.

**Please consider some other limitations below:**

1. The findings not fully replicate the complexities of natural field condition because of study only based on under controlled condition that might be potentially limiting the generalizability of the results.

We appreciate your astute observation regarding the potential limitation of our study due to its conduct under controlled conditions and the possible deviation from the complexities of natural field conditions.

We understand that the controlled environment in our study may not fully replicate all the aspects of the natural field. However, the controlled conditions were necessary in the initial stage to isolate and investigate specific factors related to the auto - toxicity of *Codonopsis pilosula*. This approach allowed us to gain a fundamental understanding of the basic mechanisms and processes involved.

We also recognize the importance of validating our findings in the natural field. In the future, we plan to conduct field - based studies to complement and expand upon our current research. By observing the growth and auto - toxic effects of *Codonopsis pilosula* in the actual field environment, we aim to enhance the generalizability of our results and provide more practical and applicable insights.

We will carefully design the field studies to account for the various factors present in the natural field, such as soil heterogeneity, climate variations, and the presence of other organisms. This will enable us to better understand how the auto - toxicity of *Codonopsis pilosula* behaves in a more complex and realistic setting.

Thank you for bringing this important issue to our attention. Your feedback will help us improve the quality and relevance of our research.

2. The study does not explore the significant interactions with other species that may be influence by allelopathic and auto-toxic effect in the context of diverse agricultural setting, while the study provides detailed insight into *C. pilosula*.

We sincerely appreciate your valuable comment. As you mentioned, in this study, we mainly focused on exploring the reasons for the auto-toxicity of *Codonopsis pilosula*. Our research group has previously investigated the continuous cropping obstacles of *C. pilosula*, and based on that, we have concentrated on understanding the auto-toxic effects within the *C. pilosula* system itself.

However, we recognize the importance of considering the interactions with other species in a diverse agricultural setting. Although this study does not directly explore the significant interactions with other species that may be influenced by allelopathic and auto-toxic effects, we understand that this is an aspect that merits further investigation.

In the future research, we will expand our scope to include the study of the interactions between *C. pilosula* and other species in different agricultural contexts. We will explore how the allelopathic and auto-toxic effects of *C. pilosula* may impact the growth and development of other plants, as well as the overall ecological balance of the agricultural system.

We believe that by conducting such research, we will be able to provide a more comprehensive understanding of the role of *C. pilosula* in the agricultural ecosystem and offer more valuable insights for sustainable agricultural practices.

Thank you again for your insightful comment, which will guide us in improving and expanding our research in the future.

3.Auto-toxic effects were tested on seed germination and early seedling growth stages, it may not for long-time effects of study.

We understand your concern that the current study only tested the auto - toxic effects on seed germination and early seedling growth stages and may not cover the long - time effects.

Our research group has previously investigated the continuous cropping obstacles of *Codonopsis pilosula*. Based on this, we have tested the auto - toxic effects on seed germination and early seedling growth stages to explore the reasons for the auto - toxicity of *Codonopsis pilosula* and identify the auto - toxic substances. Subsequently, we will conduct long - term studies to verify the long - term effects.

Thank you for your valuable comment.

4.The language of the manuscript's writings could be more polished for readability and flow, as well as reducing similarities levels.

Thank you very much for your valuable comment. We have sought the assistance of professional language editors to further refine the language and improve the overall quality of the manuscript. We are confident that the revised version has significantly improved in terms of readability, flow, and originality.
