# Supplementary material for: Allelopathic and auto-toxic effects of aqueous extracts of Codonopsis pilosula root secretions
Source: PLoS One. 2025 Jan 31;20(1):e0315657. doi: 10.1371/journal.pone.0315657 (PMC11785337; doi:10.1371/journal.pone.0315657)
Supplement: Attachment — Submitted filename: Response to Reviewers.docx [file pone.0315657.s002.docx]

Response to Reviewers

To Reviewers:

Thank you for your letter and the constructive comments on this article in your busy schedule. All of us authors have carefully read the comments that you have given us, and have discussed and revised each of these issues. The following is my list of revisions. In addition. we have resubmitted a new manuscript in the revised state, with the revisions highlighted in red. If there are any incorrect answers or questions in the manuscript. please do not hesitate to let us know.

1.Italicize Scientific Names:

Ensure C. pilosula and Codonopsis pilosula are italicized on lines 64, 65, 248, 249, 273, 274, 287, and 307.For genus names, Codonopsis should be italicized, especially on lines 229 and 230.

Thank you very much for your detailed and constructive review comments. We have carefully addressed the issues regarding the italicization of scientific names as follows:

We have gone through the manuscript and italicized C. pilosula and Codonopsis pilosula on lines 64, 65, 248, 249, 273, 274, 287, and 307. Additionally, we have ensured that the genus name Codonopsis is italicized on lines 229 and 230 as per your suggestion. This will make the presentation of scientific names more consistent with the standard scientific writing format, and it will also enhance the clarity and professionalism of the manuscript.

Once again, we appreciate your time and effort in reviewing our manuscript, and your comments have significantly improved the quality of our work.

2.Uniformity in Units (ml to mL):

Standardize "ml" to "mL" for all occurrences, particularly on lines 126 and 244, according to journal formatting preferences.

Thank you for your meticulous review. We have thoroughly revised the manuscript to address the issue of unit uniformity. We have carefully searched for all instances of "ml" and standardized them to "mL" throughout the text, especially on lines 126 and 244 as you pointed out. This ensures that our manuscript complies with the journal's formatting preferences and maintains a high level of consistency in the presentation of units.

Your feedback has been invaluable in improving the quality of our work, and we are grateful for your attention to detail.

3.Correct Capitalization in Species Names:

Adjust Angelica Sinensis on line 241 to Angelica sinensis, where the genus starts with a capital letter and the species name is lowercase.

Thank you for your valuable comment. We have revised the capitalization of the species name as you suggested. On line 241, we have changed "Angelica Sinensis" to "Angelica sinensis" to follow the correct naming convention where the genus is capitalized and the species name is in lowercase. This adjustment will enhance the scientific accuracy and consistency of our manuscript.

Thank you again for your careful review, which has helped improve the quality of our work.

4.Similarities level:

Minimize the observable similarities as much as possible.

Thank you for bringing to our attention the issue of similarity between the results and conclusion sections. We have promptly taken measures to resolve this problem.

We have re-evaluated the content of both sections. In the conclusion part, we have rephrased the statements to make them more interpretative and avoid simple repetition of the results. We have minimized the observable similarities as much as possible.

Once again, thank you for your feedback, which is instrumental in enhancing the quality and clarity of our manuscript.
